# A Hybrid Sparse Representation Model for Image Restoration

**DOI:** 10.3390/s22020537

**Published:** 2022-01-11

**Authors:** Caiyue Zhou, Yanfen Kong, Chuanyong Zhang, Lin Sun, Dongmei Wu, Chongbo Zhou

**Affiliations:** 1 School of Cyber Science and Engineering, Qufu Normal University, Qufu 273165, China; shmily961016@163.com (C.Z.); zcbsunny@hrbeu.edu.cn (L.S.); wudm@qfnu.edu.cn (D.W.); 2 Department of Information Engineering, Weihai Ocean Vocational College, Rongcheng 264300, China; kongyanfen@whovc.edu.cn (Y.K.); zhangchuanyong@whovc.edu.cn (C.Z.)

**Keywords:** image restoration, sparse representation, nonlocal self-similarity, alternating direction multiplier method

## Abstract

Group-based sparse representation (GSR) uses image nonlocal self-similarity (NSS) prior to grouping similar image patches, and then performs sparse representation. However, the traditional GSR model restores the image by training degraded images, which leads to the inevitable over-fitting of the data in the training model, resulting in poor image restoration results. In this paper, we propose a new hybrid sparse representation model (HSR) for image restoration. The proposed HSR model is improved in two aspects. On the one hand, the proposed HSR model exploits the NSS priors of both degraded images and external image datasets, making the model complementary in feature space and the plane. On the other hand, we introduce a joint sparse representation model to make better use of local sparsity and NSS characteristics of the images. This joint model integrates the patch-based sparse representation (PSR) model and GSR model, while retaining the advantages of the GSR model and the PSR model, so that the sparse representation model is unified. Extensive experimental results show that the proposed hybrid model outperforms several existing image recovery algorithms in both objective and subjective evaluations.

## 1. Introduction

The purpose of image restoration is to reconstruct high-quality images x from the degraded images y. This is a typical inverse problem, and its mathematical expression is

(1) y=Hx+n

where H denotes the degenerate operator and n is usually assumed to be zero-mean Gaussian white noise. Under different settings, Equation (1) can represent different image processing tasks. When H denotes the identity matrix, Equation (1) represents the image denoising task [1,2]; when H denotes a diagonal matrix with diagonal 1 or 0, Equation (1) represents an image inpainting task [3,4]; when *H* denotes the blurring operator, Equation (1) represents an image deblurring task [5,6]. In this paper, we focus on the image restoration task.

In order to obtain high-quality reconstructed images, image prior knowledge is usually used to regularize the solution space. In general, image restoration can be expressed as the following minimization problems:

(2) $$\hat{x}=\arg\underset{x}{\min}\frac{1}{2}{\left\|y-Hx\right\|}_{2}^{2}+\lambda R(x)$$

where the first term $\frac{1}{2}{\left\|y-Hx\right\|}_{2}^{2}$ represents data fidelity, the second term R(x) depends on the image prior, and λ is a regularization parameter that balances the two terms. Due to the ill-posed nature of image restoration, the prior knowledge of image plays an important role in improving the performance of the image restoration algorithm. In the past decades, various image prior models have been proposed, such as total variation [7], sparse representation [3,8,9,10,11], and deep convolutional neural network (CNN) [2,12,13].

Sparse representation is a commonly used technique in image processing. Sparse representation models are usually divided into two categories: analytical sparse representation models [14,15] and synthetic sparse representation models [3]. The analytic sparse representation model represents the signal by multiplying it with an analytic over-complete dictionary to produce a sparse effect. In this paper, we mainly study the synthetic sparse representation model. Generally speaking, synthetic sparse representation models in image processing can be further divided into two categories: patch-based sparse representation (PSR) [16,17] and group-based sparse representation (GSR) [3,9,10,11]. The PSR model assumes that each patch of an image can be modeled perfectly by sparse linear combination of learnable dictionaries, which are usually learned from images or image datasets. Compared with traditional analysis dictionaries, such as discrete cosine variation and wavelet, dictionaries that learn directly from images can improve sparsity and are superior in adapting to the local structure of images. For example, K-SVD based dictionary learning [17] not only shows good image denoising effects, but also has been extended to many image processing and computer vision tasks [18,19]. However, the PSR model uses an over-complete dictionary, which usually produces poor visual artifacts in image restoration [20]. Moreover, the PSR model ignores the correlation between similar patches [3,21], which usually leads to image degradation.

Inspired by the success of nonlocal self-similarity prior (NSS) [22], the GSR model was proposed. The GSR model uses patch group instead of image patch as the basic unit of image processing in sparse representation and shows great potential in various image processing tasks [3,8,9,11,23,24,25,26,27]. Dabov et al. [27] proposed the BM3D method combining transform domain filtering with NSS prior, which is still one of the most effective denoising methods. Elad et al. [23] proposed an image denoising algorithm based on the improved KSVD learning dictionary and non-local self-similarity, which combined the correlation coefficient matching criterion with the dictionary clipping method. Mairal et al. [28] proposed to learn simultaneous sparse coding (LSSC) for image restoration, improving the recovery performance of KSVD [17] through GSR. Zhang et al. [24] used non-locally similar patches as data samples and estimated statistical parameters based on PCA training. Zhang et al. [3] proposed a group-based sparse representation model for image restoration, which is essentially equivalent to a low-rank minimization model. Dong et al. [25] developed structured sparse coding with Gaussian-scale mixture prior for image restoration. Zha et al. [8] proposed a joint model to integrate the PSR model and GSR model, making image restoration establish a unified model in the field of sparse representation. Wu et al. [11] proposed structured analysis sparsity learning (SASL), which combines the structured sparse priors learned from the given degraded image and reference images in an iterative and trainable manner. Zha et al. [9] introduced the group sparse residual constraint, trying to further define and simplify the image restoration problem by reducing the group sparse residual. Zha et al. [26] proposed an image recovery method using NSS priors of both internal and external image data to develop the GSR model. Despite the great success of the GSR models in various image restoration tasks, the image restored by the traditional GSR model is prone to over-smooth effect [29]. At the same time, the traditional GSR model and various improved models only consider using the patch group of degraded image to minimize the approximate error, which will produce the effect of image over-fitting, especially when the degraded image is highly damaged.

**Figure 1:**
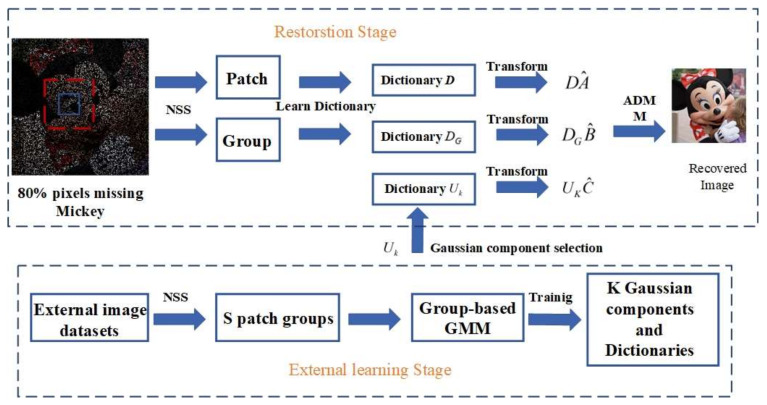
HSR-based image restoration.

Therefore, we propose a hybrid sparse representation model. The model uses both degraded image and the NSS prior of external image dataset to perform image restoration more effectively. On this basis, a joint sparse representation model is introduced. This model integrates the PSR model and GSR model into one model, which not only retains the advantages of the PSR model and the GSR model, but also reduces their shortcomings, so that the models in the sparse representation field are unified. For the convenience of description, the proposed hybrid sparse representation model is called HSR model. The NSS priors of degraded images are called internal NSS priors, and the NSS priors of external image datasets are called external NSS priors. Figure 1 shows how the HSR model can repair degraded images. The contributions of this paper are summarized as follows:

(1) We propose a hybrid sparse representation model that combines the NSS priori of degraded images and external image dataset to make full use of the specific structure of degraded image and the common characteristics of natural image;

(2) The introduction of joint model into the HSR not only retains the advantages of the PSR model and GSR model, but also alleviates their respective disadvantages.

The rest of this paper is organized as follows. Section 1 describes the related work of sparse representation. Section 2 introduces how to learn NSS prior from external image corpus. Section 3 introduces the proposed mixed sparse representation model. Section 4 employs an iterative algorithm based on the alternating direction multiplier framework to solve the proposed model. Section 5 presents the experimental results. Section 6 concludes the paper.

## 2. Fundamentals of Image Analysis Methods

This section introduces the knowledge of the HSR model. The proposed HSR model uses the NSS prior knowledge of both degraded images and external datasets and introduces a joint model that integrates the PSR model and GSR model. Therefore, the proposed HSR model is based on GSR and PSR. A brief introduction of these two models is given below.

### 2.1. Patch-Based Sparse Representation

The basic unit of patch-based sparse representation (PSR) model is image patch. Given an image $x\in R^{N}$ and a dictionary $D\in R^{b\times M}$, b≤M, where M represents the number of atoms in the dictionary D. The dictionary D in the PSR model is shared. $x_{i}=R_{i}x,\forall i=1,2,\ldots ,n$ represents the size image patch of $\sqrt{b}\times \sqrt{b}$ extracted from the position i, and $R_{i}$ represents the extraction operation. The sparse representation of each patch $x_{i}$ is to find the sparse vectors $A_{i}$ with most coefficients zero, that is $x_{i}=DA_{i}$. The $l_{0}$-norm represents the number of non-zero elements in a vector. To regularize the parameter matrix $A_{i}$ with $l_{0}$-norm is to expect most of the elements of $A_{i}$ to be 0 and the parameter $A_{i}$ to be sparse. Therefore, by solving the following $l_{0}$-norm minimization problem, each patch $x_{i}$ can be sparsely represented as

(3) $${\hat{A}}_{i}=\arg\underset{A_{i}}{\min}\frac{1}{2}{\left\|x_{i}-DA_{i}\right\|}_{2}^{2}+\lambda {\left\|A_{i}\right\|}_{0}\forall i$$

where ${\left\|\right\|}_{2}$ denotes $l_{2}$-norm and λ is a regularization parameter. In the image restoration task, the input degraded image $y\in R^{b}$ is used because the original image is not available. Extracting image patch $y_{i}$ from degraded images y, each image patch $y_{i}$ can be sparsely represented as

(4) $${\hat{A}}_{i}=\arg\underset{A_{i}}{\min}\frac{1}{2}{\left\|y_{i}-DA_{i}\right\|}_{2}^{2}+\lambda {\left\|A_{i}\right\|}_{0}\forall i$$

In this way, the whole image can be sparsely represented by a set of sparse codes ${\left\{{\hat{A}}_{i}\right\}}_{i=1}^{n}$.

### 2.2. Group-Based Sparse Representation

Compared with typical PSR models, the GSR model uses patch group as the basic unit of image processing and can produce more promising results in various image processing tasks [3,21,25]. In this subsection, we briefly introduce the GSR model.

Firstly, the image x is divided into n overlapped patch of size $\sqrt{b}\times \sqrt{b},i=1,2,\ldots ,n$. For each exemplar patch $x_{i}$, the m most similar matching patches are selected from the search window of size is W×W by the k-nearest neighbor (KNN) method to form the set $S_{G_{i}}$. Then, all patches in $S_{G_{i}}$ are stacked into a matrix $X_{i}\in R^{b\times m}$, with each patch in the collection $S_{G_{i}}$ as a column of the matrix $X_{i}$, that is $X_{i}=\{x_{i,1},x_{i,2},\ldots ,x_{i,m}\}$. Since $X_{i}$ is a matrix of all image blocks with similar structures, it is called a patch group, where $x_{i,j}$ represents the *j*-th similar patch in the *i*-th patch group. Finally, given a dictionary $D_{i}\in R^{b\times K}$, which D is usually learned from each image group, then each patch group $X_{i}$ can be sparsely represented as

(5) $${\hat{B}}_{i}=\arg\underset{B_{i}}{\min}\frac{1}{2}{\left\|X_{i}-D_{i}B_{i}\right\|}_{2}^{2}+\rho {\left\|B_{i}\right\|}_{0}\forall i$$

where $B_{i}$ represents the group sparsity coefficient of each image group, ${\left\|\right\|}_{0}$ represents the $l_{0}$-norm, and calculate the non-zero items of each column in $B_{i}$.

In image restoration tasks, since the original image is not available, we can only use the input degraded image $y\in R^{b}$. According to the above steps, the image patch $y_{i}$ are extracted from the degraded image y, search for similar matching patches to generate an image group $Y_{i}\in R^{b\times m}$, i.e., $Y_{i}=\{y_{i,1},y_{i,2},\ldots ,y_{i,m}\}$.

(6) $${\hat{B}}_{i}=\arg\underset{B_{i}}{\min}\frac{1}{2}{\left\|Y_{i}-D_{i}B_{i}\right\|}_{2}^{2}+\rho {\left\|B_{i}\right\|}_{0}\forall i$$

The entire image can be sparsely represented by groups of sparse codes ${\left\{{\hat{B}}_{i}\right\}}_{i=1}^{n}$. In the above introduction, $y_{i}$ in the PSR model and $Y_{i}$ in the GSR model are extracted from the same degraded image y.

## 3. Learning NSS Priors from External Image Datasets

As mentioned earlier, the traditional sparse representation model only uses the NSS prior of degraded image and ignores the NSS prior of external dataset. In this section, we use the group-based Gaussian mixture model (GMM) [26,30] to learn the external NSS prior from the patch group of a given training image dataset. The following briefly introduces how to learn NSS priors from external image data sets.

### 3.1. Gaussian Component Learning Based on Group GMM

Similar to the construction process of patch groups in Section 2.2, patch groups are extracted from the given external training image dataset, and each patch group is expressed as

(7) $$E_{i}={\left\{e_{i,j}\right\}}_{j=1}^{d},i=1,2,\ldots ,S$$

where $e_{i,j}$ represents the *j*-th non-local similar patch of the *i*-th patch group $E_{i}$, ∀j=1,2,…,d. In this paper, the GMM model is used to learn k Gaussian components $\left\{N(u_{k},\sum_{k})\right\}$ from the patch group ${\left\{E_{i}\right\}}_{i=1}^{S}$ of the external image dataset, and all patches in each patch group are required to belong to the same Gaussian component. The likelihood of a patch group ${\left\{E_{i}\right\}}_{i=1}^{S}$ can be expressed as

(8) $$P(E_{i})=\sum_{k=1}^{K}\pi_{k}\prod_{j=1}^{d}N(e_{i.j}|\mu_{k},\sum_{k})$$

where K is the total number of Gaussian components, $\mu_{k}$ is the mean value, $\sum_{k}$ is the covariance matrix, $\pi_{k}$ is the weight of Gaussian components, and $\sum_{k=1}^{K}\pi_{k}=1$. The GMM model is parameterized by mean vectors $\left\{\mu_{k}\right\}$, covariance matrices $\left\{\sum_{k}\right\}$, and the weights of Gaussian components $\left\{\pi_{k}\right\}$. To facilitate representation, we introduce variables $ϒ={\left\{\mu_{k},\sum_{k},\pi_{k}\right\}}_{k=1}^{K}$. Assume that all patch groups are independent, and the overall objective likelihood function is $L=\prod_{i=1}^{S}P(E)$. Taking the log of it, to maximize the objective function of using group-based GMM learning,

(9) $$InL=\sum_{i=1}^{S}In\left(\sum_{k=1}^{K}\pi_{k}\prod_{j=1}^{d}N(e_{i,j}|\mu_{k},\sum_{k})\right)$$

We can optimize Equation (9) by using the expectation maximization (EM) algorithm [30,31,32]. In the E-step, the posterior probability of the k component calculated by Bayesian formula is

(10) $$P(k|e_{i.j},ϒ)=\frac{\pi_{k}\prod_{j=1}^{d}N(e_{i,j}|u_{k},\sum_{k})}{\sum_{i=1}^{K}\pi_{l}\prod_{j=1}^{d}N(e_{i,j}|u_{l},\sum_{l})}$$

(11) $$S_{k}=\sum_{i=1}^{S}P(k|e_{i,j},ϒ)$$

In the M-step, for each patch group $E_{i}$, we update the model parameters as follows

(12) $$\pi_{k}=S_{k}/S$$

(13) $$\mu_{k}=\frac{\sum_{i=1}^{S}\pi_{k}\sum_{j=1}^{d}e_{i,j}}{\sum_{i=1}^{S}\pi_{k}}$$

(14) $$\sum_{k}=\frac{\sum_{i=1}^{S}P(k|e_{i,j},ϒ)\sum_{j=1}^{d}e_{i,j}e_{i,j}^{T}}{S_{k}}$$

By iteratively alternating between the E-Step and M-Step, the model parameters are iteratively updated until convergence is achieved.

### 3.2. Gaussian Component Selection

For patch group $Y_{i}$ of degraded image y, we can select the most appropriate Gaussian component from the training GMM. According to [31], assuming that the image is broken by a Gaussian white noise with a variance of $\sigma_{e}^{2}$, the covariance matrix of the *k*-th Gaussian component will be expressed as $\Sigma_{k}+\sigma_{e}^{2}I$, where I is the unit matrix. The *k*-th Gaussian component belonging to the image group $Y_{i}$ can be selected by posterior probability

(15) $$P(k|Y_{i})=\frac{\prod_{j=1}^{d}N(y_{i,j}|0,\sum_{k}+\sigma_{e}^{2}I)}{\sum_{l=1}^{K}\prod_{j=1}^{d}N(y_{i,j}|0,\sum_{l}+\sigma_{e}^{2}I)}$$

By maximizing Equation (15), the *k*-th Gaussian component with the highest probability can be selected for each group $Y_{i}$. Each group $E_{i}$ has the same Gaussian distribution. The covariance matrix of the *k*-th Gaussian component is denoted by $\sum_{k}$. By using the eigenvalue factorization to $\sum_{k}$, we have:

(16) $$\sum_{k}=U_{k}\Lambda_{k}U_{k}^{T}$$

where $U_{k}$ represents the orthogonal matrix composed of the eigenvector $\sum_{k}$ and the diagonal matrix $\Lambda_{k}$ of the eigenvalues. Based on the above GMM learning, the feature vector of $U_{k}$ can represent the statistical structure of NSS changes of natural images, so $U_{k}$ can be used to represent the structural changes of image groups in this component [33]. Finally, we select the best matched $U_{k}$ for each patch group $Y_{i}$. Since solving the $l_{0}$-norm minimization problem is an NP-hard problem, the $l_{0}$-norm minimization in Equation (6) is replaced by a non-convex $l_{1}$-norm. The sparse model based on external NSS can be expressed as follows

(17) $${\hat{C}}_{i}=\arg\underset{C_{i}}{\min}(\frac{1}{2}{\left\|Y_{i}-U_{k}C_{i}\right\|}_{2}^{2}+\omega {\left\|C_{i}\right\|}_{1}),\forall i$$

where $C_{i}$ represents the sparse coefficient of the *i*-th image group $Y_{i}$, and ω represents a non-negative constant. After obtaining all the sparse codes ${\left\{{\hat{C}}_{i}\right\}}_{i=1}^{n}$, a high-quality reconstructed image $\hat{x}$ can be obtained.

## 4. The Proposed Hybrid Sparse Representation Model

As mentioned above, the traditional sparse representation model only uses the internal NSS priors of degraded images, which leads to over-fitting in the image restoration process. Therefore, this paper uses both the internal NSS priors of degraded images and the external NSS priors of external image dataset. At the same time, the PSR model usually produces some undesirable visual artifacts, and the GSR model leads to over-smoothing effects in various image processing tasks. In order to overcome their shortcomings and improve the image restoration effect, we have introduced a joint model [8] based on both internal and external NSS priors. This model integrates the PSR model and GSR model, instead of using Equations (4) and (6) separately. Combining Equations (4), (6) and (17), the proposed new hybrid sparse representation model is expressed as

(18) $$\begin{array}{c} ({\hat{A}}_{i},{\hat{B}}_{i},{\hat{C}}_{i})=\arg\underset{A_{i},B_{i},C_{i}}{\min}\left(\frac{1}{2\mu }{\left\|Y_{i}-L_{i}N_{i}\right\|}_{2}^{2}+\frac{1}{2\eta }{\left\|Y_{i}-U_{k}C_{i}\right\|}_{2}^{2}+\tau {\left\|A_{i}\right\|}_{0}+\varphi {\left\|B_{i}\right\|}_{0}+\omega {\left\|C_{i}\right\|}_{1}\right)\\ L_{i}=[\begin{array}{cc} D & D_{i} \end{array}],\;N_{i}=\left[\begin{array}{c} A_{i}\\ B_{i} \end{array}\right] \end{array}$$

where $N_{i}$ represents the internal sparse coefficient of the joint sparse representation model, and $L_{i}$ represents the internal joint dictionary of the joint sparse representation model. $U_{k}$ represents the external dictionary, which is learned from the image group of the external image data set using the external NSS prior [26,30]. μ and η represent non-zero constants and act as balance factors to make the solution of Equation (18) more feasible. $\tau =\frac{\lambda }{2}$, $\varphi =\frac{\rho }{2}$, ω represents the regularization parameter, which is used to balance the sparse coefficients terms of ${\left\|A_{i}\right\|}_{0}$, ${\left\|B_{i}\right\|}_{0}$, and ${\left\|C_{i}\right\|}_{1}$. The sparse coefficient $A_{i}$ corresponds to the sparseness of the image patch on the basis of maintaining the local consistency of the image, which reduces the over-smoothing effect. The sparse coefficient $B_{i}$ corresponds to the sparseness of patch group on the basis of maintaining the non-local consistency of the image and suppresses the undesirable visual artifacts. For specific details of the joint sparse representation model, please refer to [8]. Based on the above analysis, the proposed hybrid sparse representation model not only uses the internal and external NSS priors, but also unifies the sparse representation model.

The hybrid sparse representation model is used in the task of image restoration, and the joint Equations (1) and (18) are expressed as

(19) $$\begin{array}{c} ({\hat{A}}_{i},{\hat{B}}_{i},{\hat{C}}_{i})=\arg\underset{A_{i},B_{i},C_{i}}{\min}\left(\frac{1}{2\mu }{\left\|y-HLN\right\|}_{2}^{2}+\frac{1}{2\eta }{\left\|y-HUC\right\|}_{2}^{2}+\tau \sum_{i=1}^{n}{\left\|A_{i}\right\|}_{0}+\varphi \sum_{i=1}^{n}{\left\|B_{i}\right\|}_{0}+\omega \sum_{i=1}^{n}{\left\|C_{i}\right\|}_{1}\right)\\ L=[\begin{array}{cc} D & D_{G} \end{array}],\;N=\left[\begin{array}{c} A\\ B \end{array}\right] \end{array}$$

In Equation (19), L represents the internal dictionary of the joint sparse representation model, and U represents the external dictionary. N represents the sparse coefficient of the joint sparse representation model, and C represents the external sparse coefficient. The hybrid sparse representation model proposed in Equation (19) not only comprehensively considers the NSS priors of internal image and external image database, which can provide mutually complementary information for image reconstruction, but also unifies the sparse representation model by combining the PSR model and GSR model.

## 5. The Solution Process of the Proposed Hybrid Sparse Representation Model

In this Section, in order to make the proposed model manageable and robust, the alternating direction method of multipliers (ADMM) [34,35] is adopted to solve the large-scale optimization problem in Equation (19). Specifically, the minimization of Equation (19) involves three sub-problems, including $A_{i}$, $B_{i}$, and $C_{i}$. Different from the traditional optimization strategies that only considers the fixed values of parameters μ, η, τ, φ, and ω, we adaptively adjust all parameters in Equation (19) at each iteration to ensure the stability and practicability of the algorithm. The specific implementation details of the hybrid sparse representation model are given below.

### 5.1. Solution of Hybrid Sparse Representation Model Based on ADMM

Equation (19) is a large-scale non-convex optimization problem. In order to make the optimization problem easy to handle, the alternating direction multiplier method (ADMM) is used. The basic principle of ADMM is to decompose the unconstrained minimization problem into different constrained sub-problems. The following is a brief introduction to ADMM algorithm through a constraint optimization problem,

(20) $$\underset{Z\in R^{N},N\in R^{M}}{\min}f(Z)+g(N),s.t.Z=LN$$

where $L\in R^{M\times N}$, $f:R^{N}\to R$, $g:R^{M}\to R$. The basic ADMM is shown in Algorithm 1, where t represents the number of iterations.
**Algorithm 1.** ADMMRequire: Z and N1: Initialize t=0, μ>0, $Z_{0}=0$, $J_{0}=0$2: for t=0 to Max-Iter do3: $Z^{t+1}=\arg\underset{Z}{\min}f(Z)+\frac{v}{2}{\left\|Z-N^{t}-J^{t}\right\|}_{2}^{2}$4: $N^{t+1}=\arg\underset{N}{\min}g(N)+\frac{v}{2}{\left\|Z^{t+1}-N-J^{t}\right\|}_{2}^{2}$5: $J^{t+1}=J^{t}-(Z^{t+1}-N^{t+1})$6: end for

Going back to the hybrid sparse representation model, we transform Equation (19) into two constraint problems and call the ADMM method to solve it. We first transform Equation (19) into an equivalent constraint form by introducing auxiliary variables *Z* and *Q*,

(21) $$\begin{array}{c} ({\hat{A}}_{i},{\hat{B}}_{i},{\hat{C}}_{i})=\arg\underset{A_{i},B_{i},C_{i}}{\min}\left(\frac{1}{2\mu }{\left\|y-HZ\right\|}_{2}^{2}+\frac{1}{2\eta }{\left\|y-HQ\right\|}_{2}^{2}+\tau \sum_{i=1}^{n}{\left\|A_{i}\right\|}_{0}+\varphi \sum_{i=1}^{n}{\left\|B_{i}\right\|}_{0}+\omega \sum_{i=1}^{n}{\left\|C_{i}\right\|}_{1}\right)\\ s.t.Z=LN,Q=UC \end{array}$$

To facilitate the solution, Equation (21) can be decomposed into two constrained optimization problems,

(22) $$({\hat{A}}_{i},{\hat{B}}_{i})=\arg\underset{A_{i},B_{i},C_{i}}{\min}\left(\frac{1}{2\mu }{\left\|y-HZ\right\|}_{2}^{2}+\tau \sum_{i=1}^{n}{\left\|A_{i}\right\|}_{0}+\varphi \sum_{i=1}^{n}{\left\|B_{i}\right\|}_{0}\right),s.t.Z=LN$$

(23) $$({\hat{C}}_{i})=\arg\underset{C_{i}}{\min}\left(\frac{1}{2\eta }{\left\|y-HQ\right\|}_{2}^{2}+\omega \sum_{i=1}^{n}{\left\|C_{i}\right\|}_{1}\right),s.t.Q=UC$$

Equation (22) represents the constrained optimization problem of solving the internal joint sparse representation model, and Equation (23) represents the constrained optimization problem of solving the external sparse representation model.

### 5.2. Solution of Internal Sparse Representation Model

Solving the internal sparse representation model in Equation (22), defining $f(Z)=\frac{1}{2\mu }{\left\|y-HZ\right\|}_{2}^{2}$, $g(N)=\tau \sum_{i=1}^{n}{\left\|A_{i}\right\|}_{0}+\varphi \sum_{i=1}^{n}{\left\|B_{i}\right\|}_{0}$, and using line 3 in Algorithm 1,

(24) $$\begin{array}{c} {\hat{Z}}^{t+1}=\arg\underset{Z}{\min}f(Z)+\frac{v}{2}{\left\|Z-N^{t}-J^{t}\right\|}_{2}^{2}\\ =\arg\underset{Z}{\min}\frac{1}{2\mu }{\left\|y-HZ\right\|}_{2}^{2}+\frac{v}{2}{\left\|Z-[\begin{array}{cc} D & D_{G} \end{array}][\begin{array}{c} A^{t}\\ B_{G}^{t} \end{array}]-J^{t}\right\|}_{2}^{2}\\ =\arg\underset{Z}{\min}\frac{1}{2\mu }{\left\|y-HZ\right\|}_{2}^{2}+\frac{v}{2}{\left\|Z-DA^{t}-D_{G}B_{G}^{t}-J^{t}\right\|}_{2}^{2} \end{array}$$

where D represents the fixed dictionary in the PSR model, and $D_{G}$ represents the cascade of all sub-dictionaries $D_{i}$ in the GSR model. Using line 4 in Algorithm 1, we obtain

(25) $$\begin{array}{c} {\hat{N}}^{t+1}=\arg\underset{N}{\min}g(N)+\frac{v}{2}{\left\|Z^{t+1}-LN-J^{t}\right\|}_{2}^{2}\\ =\arg\underset{N}{\min}\tau \sum_{i=1}^{n}{\left\|A_{i}\right\|}_{0}+\varphi \sum_{i=1}^{n}{\left\|B_{i}\right\|}_{1}+\frac{v}{2}{\left\|Z^{t+1}-[\begin{array}{cc} D & D_{G} \end{array}][\begin{array}{c} A\\ B_{G} \end{array}]-J^{t}\right\|}_{2}^{2}\\ =\arg\underset{N}{\min}\tau \sum_{i=1}^{n}{\left\|A_{i}\right\|}_{0}+\varphi \sum_{i=1}^{n}{\left\|B_{i}\right\|}_{1}+\frac{v}{2}{\left\|Z^{t+1}-DA-D_{G}B_{G}-J^{t}\right\|}_{2}^{2} \end{array}$$

The minimization problem N in Equation (25) is decomposed into $A_{i}$ and $B_{i}$, and solved respectively, as

(26) $${\hat{A}}_{i}^{t+1}=\arg\underset{A_{i}}{\min}\tau \sum_{i=1}^{n}{\left\|A_{i}\right\|}_{0}+\frac{v}{2}{\left\|Z^{t+1}-DA-D_{G}B_{G}-J^{t}\right\|}_{2}^{2}$$

(27) $${\hat{B}}_{i}^{t+1}=\arg\underset{B_{i}}{\min}\varphi \sum_{i=1}^{n}{\left\|B_{i}\right\|}_{1}+\frac{v}{2}{\left\|Z^{t+1}-DA-D_{G}B_{G}-J^{t}\right\|}_{2}^{2}$$

Finally, using line 5 of Algorithm 1 to update $J^{t}$,

(28) $$J^{t+1}=J^{t}-(Z^{t+1}-DA^{t+1}-D_{G}B_{G}^{t+1})$$

In summary, the minimization of Equation (22) involves three minimization problems, including Z, $A_{i}$, and $B_{i}$. Equation (26) represents the PSR model, and Equation (27) represents the GSR model. The implementation details of an effective solution to each sub-problem are given below.

#### 5.2.1. Solution of Sub-Problem Z

Given A and $B_{G}$, the sub-problem Z in Equation (24) is transformed into,

(29) $$\underset{Z_{i}}{\min L_{1}(Z_{i})}=\underset{Z_{i}}{\min}\sum_{i=1}^{n}\frac{1}{2\mu }{\left\|Y_{i}-H_{i}Z_{i}\right\|}_{2}^{2}+\frac{v}{2}{\left\|Z_{i}-DA_{i}-D_{i}B_{i}-J_{i}\right\|}_{2}^{2},\forall i$$

Equation (29) is a quadratic form that has a closed-form solution so that

(30) $${\hat{Z}}_{i}={\left(H_{i}^{T}H_{i}+v\mu I\right)}^{-1}(H_{i}^{T}Y_{i}+v\mu (DA_{i}+D_{i}B_{i}+J_{i})),\forall i$$

In Equation (30), I denotes the unit matrix of the desired dimension, and $J_{i}$ is the corresponding element in J. In Equation (30), (26) and (27) are used in combination to estimate each.

#### 5.2.2. Solution of Sub-Problem $A_{i}$

For each image patch in Equation (26), the sub-problem can be re-expressed as

(31) $$\underset{A_{i}}{\min}L_{2}(A_{i})=\underset{A_{i}}{\min}\sum_{i=1}^{n}\frac{1}{2}{\left\|DA_{i}-r_{i}\right\|}_{2}^{2}+\frac{\mu \tau }{v}{\left\|A_{i}\right\|}_{0},\forall i$$

where $r_{i}=Z_{i}-D_{i}B_{i}-J_{i}$. Equation (31) is a sparse representation problem, where the constraint form is directly solved,

(32) $$\underset{A_{i}}{\min}{\left\|A_{i}\right\|}_{0},s.t.{\left\|DA_{i}-r_{i}\right\|}_{2}^{2}\leq \theta ,\forall i$$

where θ represents a small constant. Equation (32) can be effectively solved by the orthogonal matching pursuit (OMP) algorithm [36].

#### 5.2.3. Solution of Sub-Problem $B_{i}$

Given Z and A, the sub-problem in Equation (27) can be transformed into,

(33) $$\underset{B_{i}}{\min}L_{2}(B_{i})=\underset{B_{i}}{\min}\sum_{i=1}^{n}\frac{1}{2}{\left\|D_{i}B_{i}-R_{i}\right\|}_{2}^{2}+\frac{\mu \varphi }{v}{\left\|B_{i}\right\|}_{1},\forall i$$

where $R_{i}=Z_{i}-DA_{i}-J_{i}$. Solving Equation (33), we find,

(34) $${\hat{B}}_{i}=\underset{B_{i}}{\arg\min}\sum_{i=1}^{n}\frac{1}{2}{\left\|R_{i}-D_{i}B_{i}\right\|}_{2}^{2}+\frac{\mu \varphi }{v}{\left\|B_{i}\right\|}_{1},\forall i$$

An important problem in solving sub-problem $B_{i}$ is the choice of dictionary $D_{i}$. To adapt the local structure of the image, a dictionary based on principal component analysis (PCA) is learned for each group $R_{i}$. Due to the orthogonality of dictionary $D_{i}$, Equation (34) can be rewritten as

(35) $${\hat{B}}_{i}=\underset{B_{i}}{\arg\min}\sum_{i=1}^{n}\frac{1}{2}{\left\|\gamma_{i}-B_{i}\right\|}_{2}^{2}+\frac{\mu \varphi }{v}{\left\|B_{i}\right\|}_{1},\forall i$$

where $R_{i}=D_{i}\gamma_{i}$. We can solve the closed solution for each $B_{i}$ by soft thresholding [31],

(36) $${\hat{B}}_{i}=soft(\gamma_{i},\frac{\mu \varphi }{v})$$

### 5.3. Solution of External Sparse Representation Model

Solving the external sparse representation model in Equation (23), defining $f(Q)=\frac{1}{2\eta }{\left\|y-HQ\right\|}_{2}^{2}$,$g(C)=\omega \sum_{i=1}^{n}{\left\|C_{i}\right\|}_{1}$, and using line 3 in Algorithm 1,

(37) $$\begin{array}{c} {\hat{Q}}^{t+1}=\arg\underset{Q}{\min}\;f(Q)+\frac{v}{2}{\left\|Q-UC^{t}-O^{t}\right\|}_{2}^{2}\\ =\arg\underset{Q}{\min}\frac{1}{2\eta }{\left\|y-HQ\right\|}_{2}^{2}+\frac{v}{2}{\left\|Q-UC^{t}-O^{t}\right\|}_{2}^{2} \end{array}$$

Using line 4 in Algorithm 1, we obtain

(38) $$\begin{array}{c} {\hat{C}}^{t+1}=\arg\underset{C}{\min}\;g(C)+\frac{v}{2}{\left\|Q^{t+1}-UC-O^{t}\right\|}_{2}^{2}\\ =\arg\underset{C_{i}}{\min}\;\omega \sum_{i=1}^{n}{\left\|C_{i}\right\|}_{0}+\frac{v}{2}{\left\|Q^{t+1}-UC-O^{t}\right\|}_{2}^{2} \end{array}$$

Finally, using line 5 of Algorithm 1 to update $O^{t}$,

(39) $$O^{t+1}=O^{t}-(Q^{t+1}-UC^{t+1})$$

In summary, the minimization of Equation (23) involves two minimization sub-problems, including Q and $C_{i}$. The solution procedure for Q and $C_{i}$ is similar to that in Section 5.2, and the implementation details of an efficient solution for each sub-problem are given below.

### 5.3.1. Solution of Sub-Problem Q

Given the internal sparse representation model, Equation (23) translates into

(40) $${\hat{C}}_{i}=\arg\underset{C_{i}}{\min}\sum_{i=1}^{n}\frac{1}{2}{\left\|Y_{i}-H_{i}Q_{i}\right\|}_{2}^{2}+\frac{\eta \varepsilon }{v}{\left\|C_{i}\right\|}_{1},s.t.Q_{i}=U_{i}C_{i}$$

Equation (37) is a quadratic form that has a closed-form solution so that

(41) $${\hat{Q}}_{i}={\left(H_{i}^{T}H_{i}+\eta \varepsilon I\right)}^{-1}(H_{i}^{T}Y_{i}+\eta \varepsilon (UC_{i}+O_{i})),\forall i$$

In Equation (38), I denotes the unit matrix of the desired dimension and $O_{i}$ is the corresponding element in O.

### 5.3.2. Solution of Sub-Problem $C_{i}$

Given Q, the sub-problem $C_{i}$ in Equation (40) can be transformed so that

(42) $${\hat{C}}_{i}=\arg\underset{C_{i}}{\min}\sum_{i=1}^{n}\frac{1}{2}{\left\|Y_{i}-U_{i}C_{i}\right\|}_{2}^{2}+\frac{\eta \varepsilon }{v}{\left\|C_{i}\right\|}_{1},\forall i$$

Evidently, Equation (42) can be viewed as a sparse representation problem for each image group $Y_{i}$. According to Section 2, we can select the best-matching Gaussian component for each group through Equation (15), and then assign the best matching PCA-based dictionary to each group according to Equation (16). Due to the orthogonality of dictionary $U_{i}$, Equation (42) can be rewritten as

(43) $${\hat{C}}_{i}=\arg\underset{C_{i}}{\min}\sum_{i=1}^{n}\frac{1}{2}{\left\|\kappa_{i}-C_{i}\right\|}_{2}^{2}+\frac{\eta \varepsilon }{v}{\left\|C_{i}\right\|}_{1},\forall i$$

where $Y_{i}=U_{i}\kappa_{i}$. We can solve the closed solution for each a by soft threshold [37],

(44) $${\hat{C}}_{i}=soft(\kappa_{i},\frac{\eta \varepsilon }{v})$$

A complete description of the hybrid sparse representation model for image restoration is given in Algorithm 2.
**Algorithm 2.** A hybrid sparse representation model for image restorationRequire degraded image y, mask H and group-based GMM1: Initialize ${\hat{x}}^{0}=y$, $A_{i}^{0}=0$, $B_{i}^{0}=0$, $C_{i}^{0}=0$2: Set parameters t, b, W, m, μ, η, τ, φ, ω, v, ς, ε3: **for** *t* = 0 to Max-Iter **do**4: Calculate $\sigma_{e}$ by Equation (45)5: Update $O^{t+1}$ by Equation (39)6: **for** Each patch group $Y_{i}$ **do**7:  Select the *k*-th optimal Gaussian component by Equation (15)8:  Select the dictionary $U_{k}$ by Equation(16)9:   Update $C_{i}^{t+1}$ by Equation (44)10: **end for**11: Update $Z^{t+1}$ by Equation (30)12:   $R^{t+1}=Z^{t+1}-D_{G}B_{G}-J^{t}$13:   Create dictionary D by $R^{t+1}$ using KSVD14:   each patch $r_{i}$ **do**15:   Update $A_{i}^{t+1}$ by Equation (32)16:   **end for**17:   $R_{G}^{t+1}=Z^{t+1}-DA-J^{t}$18:   **for** each patch group $R_{i}$ **do**19:   Create dictionaries $D_{G}$ by $R_{i}^{t+1}$ using PCA20:   Update $B_{i}^{t+1}$ by Equation (36)21:   **end for**22:   Update $C^{t+1}$ by concatenating all $C_{i}$23:   Update
$A^{t+1}$ by concatenating all $A_{i}$24:   Update $B^{t+1}$ by concatenating all $B_{i}$25:   Update $D^{t+1}$ by concatenating all $D_{i}$26: **end for**27: Output: The final restored image $\hat{x}=x^{t+1}$.

### 5.4. Adaptive Parameter Adjustment Strategy

There are six parameters in Equation (21), namely μ, η, τ, φ, ω, and v. A fixed value is usually chosen for each parameter based on experience. However, this makes it difficult to guarantee the stability and effectiveness of the whole algorithm. To address this problem, an adaptive parameter adjustment scheme is proposed to make the proposed algorithm more stable and practical. An iterative regularization strategy [38] is used to update the estimate of the noise variance $\sigma_{e}$. The standard deviation of the noise $\sigma_{e}$ at the *t*-th iteration is expressed as

(45) $$\sigma_{e}^{t}=\delta \sqrt{\sigma_{e}^{2}-{\left\|\hat{x}-y\right\|}_{2}^{2}}$$

where t denotes the number of iterations, δ denotes the scale factor controlling the variance estimation, and the scheme has been widely used for image denoising with Gaussian noise variance estimation [30,38].

Therefore, $\mu^{t}$ and $\eta^{t}$ can be expressed as

(46) $$\mu^{t}=a{\left(\sigma_{e}^{2}\right)}^{t}$$

(47) $$\eta^{t}=b{\left(\sigma_{e}^{2}\right)}^{t}$$

where $\gamma_{i}$ and $\kappa_{i}$ denote the estimated standard deviation of ${\hat{B}}_{i}$ and ${\hat{C}}_{i}$ [39], respectively. ς denotes a constant with a small value to avoid division by zero. In order to make the proposed algorithm more accurate and practical, according to [37], in the *t*-th iteration, the ADMM balance factor a is set to

(48) $$v^{t}=\frac{1}{c{\left(\sigma_{e}^{2}\right)}^{t}}$$

where c denotes the scale factor.

## 6. Experimental Results

**Figure 2:**
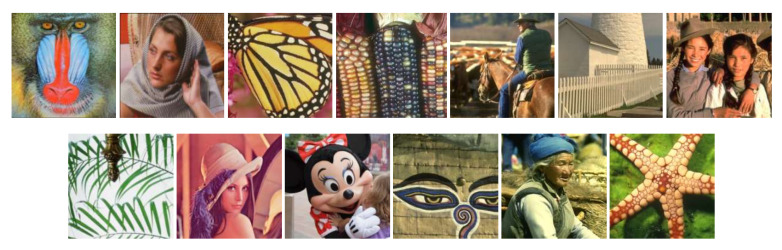
Test images.

In this section, the experimental results of the proposed HSR model and seven comparison methods are given, including the SALSA [40], BPFA [41], GSR [3], JSR-SR [8], GSRC-NLP [9], IR-CNN [42], and IDBP [43] methods. All experiments were carried out on Intel (R) Core (TM) I7-6700 CPU and 3.40 GHz CPU PC under Matlab 2018B environment. The source code for all competing methods is open source, and we use the default parameter settings. The 13 images used for the experimental tests are shown in Figure 2. In order to evaluate the quality of the restored images, an experimental comparative analysis of the restored images was performed from both objective and subjective aspects. For objective evaluation, the peak signal to noise ratio (PSNR) and structural similarity (SSIM) [44] metrics were used for the experimental comparison of the restored images. The PSNR was calculated as shown in Equations (49) and (50),

(49) $$MSE=\frac{1}{H\times W}\sum_{i=0}^{H-1}\sum_{j=0}^{W-1}{\left\|X(i,j)-Y(i,j)\right\|}^{2}$$

(50) $$PSNR=10\cdot \log_{10}(\frac{{\left(2^{n}-1\right)}^{2}}{MSE})$$

where X and Y denote the original image and the restored image, respectively, and H×W denotes the size of the image. Equation (20) is used to calculate the mean squared error MSE of the original image X and the restored image Y. Equation (50) is the calculation formula of PSNR, and n is the number of bits per pixel. A larger value of PSNR indicates less image distortion. The calculation of SSIM is shown in Equations (51)–(55),

(51) $$l(X,Y)=\frac{2u_{X}u_{Y}+C_{1}}{u_{X}^{2}+u_{Y}^{2}+C_{1}},\;c(X,Y)=\frac{2\sigma_{X}\sigma_{Y}+C_{2}}{\sigma_{X}^{2}+\sigma_{Y}^{2}+C_{2}},\;s(X,Y)=\frac{2\sigma_{XY}+C_{3}}{\sigma_{X}\sigma_{Y}+C_{3}}$$

(52) $$u_{X}=\frac{1}{H\times W}\sum_{i=1}^{H}\sum_{j=1}^{W}X\left(i,j\right)$$

(53) $$\sigma_{X}^{2}=\frac{1}{H\times W-1}\sum_{i=1}^{H}{\sum_{j=1}^{W}\left(X\left(i,j\right)-u_{X}\right)}^{2}$$

(54) $$\sigma_{XY}=\frac{1}{H\times W-1}\sum_{i=1}^{H}\sum_{j=1}^{W}\left((X(i,j)-u_{X})(Y(i,j)-u_{Y})\right)$$

(55) SSIM(X,Y)=l(X,Y)c(X,Y)s(X,Y)

In Equation (51), SSIM measures similarity in terms of luminance l, contrast c, and image structure s. Where $u_{X}$ and $u_{Y}$ denote the mean of the original image X and the restored image Y of size H×W, respectively; $\sigma_{X}$ and $\sigma_{Y}$ denote the variance of the original image X and the restored image Y, respectively; and $\sigma_{XY}$ denotes the covariance of the original image X and the restored image Y. $C_{1}$, $C_{2}$, and $C_{3}$ are constants and introducing a constant can avoid the situation where the denominator is 0. The SSIM indicator is closer to human subjective feelings, and its value range is $\left[0,1\right]$. The larger the value of SSIM, the more similar the two images are, and the better the effect of image restoration.

For color images, this paper only focuses on the restoration of the luminance channel in YCrCb space. In the group-based GMM learning phase, the training patch group used in the experiment is collected from the Kodak photoCD dataset, which includes 24 natural images.

### 6.1. Objective Evaluation

In the image restoration task, the image restoration results are given for four masks, i.e., 80%, 70%, 60%, and 50% of random pixel loss. The parameters of the HSR model used for image restoration are set as follows: the search window W×W is set to 25×25, the size of the image patch is set to 8×8, the number of similar patches is set to 60, $\sigma_{e}=\sqrt{2}$, $\varsigma =e^{-14}$, and v=0.2. We compared the proposed HSR model with seven restoration methods, including SALSA [40], BPFA [41], GSR [3], JPG-SR [8], GSRC-NLP [9], IR-CNN [42] and IDBP [43]. Among these seven methods for comparison, SALSA [40], BPFA [41], GSR [3], JPG-SR [8], and GSRC-NLP [9] are based on traditional image restoration algorithms. The GSR [3], JPG-SR [8], and GSRC-NLP [9] methods are image restoration algorithms based on the traditional GSR model, which belong to the same type of model as our proposed HSR model. SALSA [40] and BPFA [41] are not based on GSR. In order to comprehensively evaluate the performance of the proposed model for image restoration, the proposed HSR model was also compared with algorithms based on deep learning [42,43].

The SALSA model [40] proposes an algorithm belonging to the augmented Lagrangian method family to deal with constraint problems. The method solves optimization problems where the optimal regularization parameters are tuned by manual trial and error, which requires considerable time and effort to achieve the optimal value of the method. The BPFA model [41] utilizes a non-parametric Bayesian dictionary learning method for image sparse representation, and uses image patches as the basic unit of sparse representation, which ignores the similarity between image patches. In terms of the average value, the proposed HSR model is 4.74 dB and 6.19 dB higher than SALAS and BPFA methods respectively.

The GSR method [3] is a typical representative of the traditional GSR model, and the JPG-SR method [8] and the GSRC-NLP method [9] are both improved methods based on the GSR model. The GSR method, the JPG-SR method and the GSRC-NLP method only utilize the internal NSS prior. However, the HSR model proposed in this paper combines internal and external NSS priors. In terms of the average value, the HSR model proposed in this paper improves 1.47 dB, 1.43 dB, and 1.06 dB over the GSR, JPG-SR, and GSRC-NLP methods respectively. IRCNN [42] and IDBP method [43] are recovery methods based on deep learning, using powerful prior knowledge of deep neural networks. In terms of the average value, the proposed HSR model improves 3.66 dB and 3.01 dB over the IRCNN and IDBP methods, respectively.

As shown in Table 1, Table 2, Table 3 and Table 4, the PSNR of the proposed HSR model on images with a pixel loss rate of 80%, 70%, 60% and 50% is higher than that of SALSA, BPFA, GSR, JRG-SR, GSRC-NLP, IR-CNN and IDBP. It can be seen from the statistical SSIM values in Table 5, Table 6, Table 7 and Table 8, that the HSR model is better than other methods in most cases. The experimental results in Table 1, Table 2, Table 3 and Table 4 and Table 5, Table 6, Table 7 and Table 8 prove that the proposed HSR model is effective and gives good restoration results compared to the comparison method.

### 6.2. Subjective Assessment

**Figure 3:**
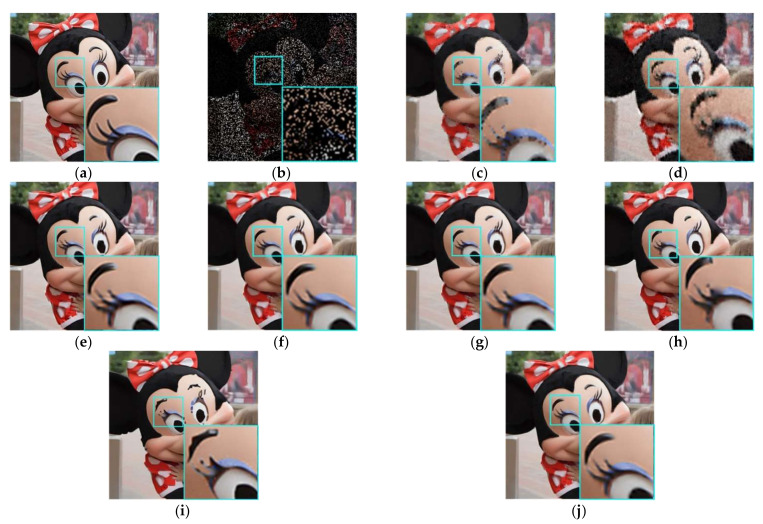
Results of Mickey image restoration with pixel missing rate of 80%. (**a**) Mickey; (**b**) Mickey with pixel loss rate 80%; (**c**) the result of SALSA [40] (PSNR = 24.26, SSIM = 0.8000); (**d**) the result of BPFA [41] (PSNR = 21.96, SSIM = 0.6512); (**e**) the result of GSR [3] (PSNR = 26.50, SSIM = 0.8816); (**f**) the result of JPG-SR [8] (PSNR = 26.75, SSIM = 0.8696); (**g**) the result of GSRC-NLP [9] (PSNR = 27.00, SSIM = 0.9197); (**h**) the result of IR-CNN [42] (PSNR = 24.87, SSIM = 0.8482); (**i**) the result of IDBP [43] (PSNR = 25.26, SSIM = 8422); (**j**) the result of our proposed HSR (PSNR = 28.25, SSIM = 8963).

The visual comparison between the proposed HSR model in this paper and SALSA [40], BPFA [41], GSR [3], JPG-SR [8], GSRC-NLP [9], IR-CNN [42] and IDBP [43] methods after restoration of the image Mickey with pixel missing rate 80% is given in Figure 3. It can be observed from Figure 3 that the SALSA [40] and BPFA [41] methods cannot recover sharp edges and fine details. The GSR [3] method is better in recovering details, but produces an over-smoothing effect. The JPG-SR [8] method can obtain better visual quality than GSR [3] method. However, the objective evaluation results in Table 1, Table 2, Table 3, Table 4 and Table 5 and Table 5, Table 6, Table 7 and Table 8 show that although the JPG-SR [8] method has a higher mean value of PSNR than the GSR [3] method in Table 1, Table 2, Table 3, Table 4 and Table 5, in the actual restoration process, the PSNR and SSIM values of some images after restoration are lower than the restoration results of GSR [3] method. The image restoration effect of the JPG-SR [8] method is unstable, and only some of the image restoration results are better than the GSR [3] method. The GSRC-NLP [9] method can obtain similar visual effects as our proposed HSR model, which is not easy to distinguish from the naked eye. However, according to the experimental results in Table 1, Table 2, Table 3, Table 4 and Table 5 and Table 5, Table 6, Table 7 and Table 8, our proposed HSR model has better objective evaluation results. The visual result of our proposed method is also better in recovering details than the results of IR-CNN [42] and IDBP [43]. The visual results in Figure 3 show that our proposed HSR model retains clear edges and details, especially at higher pixel missing rates, and produces the result with the best visual quality.

### 6.3. Running Time

In this section, we present a comparison of the proposed HSR method with other comparison methods in terms of running time. Taking image Butterfly as an example, the running time of all comparison methods is compared when the pixel loss rate is 50%. As can be seen from Table 9, the processing time of HSR method proposed in this paper is 5000.22 s for the image, which is less than 5027.67 s of the GSRC-NLP method. The proposed HSR method utilizes NSS to construct internal and external image groups and needs to learn the corresponding dictionaries, which requires higher computational workload and therefore consumes more time. To reduce processing time in our future work, learning external NSS priors from the external data set will be done in advance in the Kodak photoCD data set. Through one-time learning from Kodak photoCD data set, the external NSS priors are obtained. The priors learned in advance are applied to speed up the proposed HSR method.

## 7. Conclusions

In order to improve the repair performance of the traditional GSR model, we propose a new hybrid sparse representation model. The model uses the NSS prior of degraded image and external image data set, so that the model is complementary in the feature space and the plane. And on this basis, we introduced a joint sparse representation model. The joint model integrates the PSR model and the GSR model, while retaining their advantages, overcoming their shortcomings, and unifying the sparse representation model. Experimental results show that the model is comparable to the test method, and it is better than several state-of-the-art image restoration and map methods in both objective and subjective aspects.

## Figures and Tables

**Table 1:** PSNR values of our proposed HSR model and other comparison models after image restoration with pixels missing rate 80%.

	Pixels Missing = 80%							
Images	SALSA	BPFA	GSR	JPG-SR	GSRC-NLP	IR-CNN	IDBP	HSR
Bahoon	24.41	23.25	24.57	25.40	25.55	23.77	25.08	**26.17**
Barbara	22.62	22.60	31.32	30.16	30.93	24.33	22.73	**31.62**
Butterfly	22.85	21.06	26.03	26.58	26.78	24.50	25.24	**28.30**
Corn	24.28	22.37	26.91	26.40	26.76	23.89	26.05	**27.39**
Cowboy	23.72	22.16	25.37	25.61	25.95	24.36	24.43	**27.54**
Fence	21.80	22.87	29.66	29.40	30.02	26.09	25.03	**30.57**
Girl	23.79	22.47	25.50	25.55	26.02	24.17	25.03	**27.13**
Leaves	22.03	19.30	27.46	27.33	27.62	23.57	25.84	**29.10**
Lena	28.20	27.56	31.41	31.25	31.86	29.53	29.69	**32.55**
Mickey	24.46	21.96	26.50	26.75	27.00	24.85	25.40	**28.25**
Mural	23.15	21.10	26.01	26.29	26.56	24.87	25.26	**27.59**
Nanna	24.12	22.38	25.24	25.92	26.17	24.68	25.51	**27.48**
Starifish	25.70	23.95	27.84	27.80	28.04	25.64	26.88	**28.99**
Average	23.93	22.54	27.22	27.26	27.64	24.94	25.55	**28.66**

**Table 2:** PSNR values of our proposed HSR model and other comparison models after image restoration with pixels missing rate 70%.

	Pixels Missing = 70%							
Images	SALSA	BPFA	GSR	JPG-SR	GSRC-NLP	IR-CNN	IDBP	HSR
Bahoon	25.71	24.57	26.17	26.80	26.98	25.17	26.41	**27.47**
Barbara	23.38	25.49	**34.43**	33.45	33.98	27.12	26.23	**34.43**
Butterfly	25.06	23.95	28.92	29.24	29.47	27.34	28.29	**30.12**
Corn	26.11	25.31	29.35	28.82	29.10	26.58	28.09	**29.63**
Cowboy	25.70	24.55	27.63	27.78	28.04	26.49	27.15	**29.33**
Fence	23.57	25.56	31.73	31.53	31.82	28.71	29.24	**32.26**
Girl	25.47	24.71	27.86	27.96	28.20	26.47	27.45	**28.99**
Leaves	24.36	22.43	31.18	30.67	30.88	27.09	28.99	**32.02**
Lena	28.82	30.36	33.54	33.40	33.85	31.98	32.21	**34.48**
Mickey	28.98	24.16	29.02	28.93	29.31	27.74	28.69	**30.14**
Mural	25.00	23.34	28.46	28.50	28.71	27.42	27.68	**29.25**
Nanna	25.44	24.47	27.89	28.24	28.51	26.90	27.24	**29.45**
Starifish	27.55	26.83	30.31	30.11	30.46	28.16	29.28	**31.09**
Average	25.78	25.06	29.73	29.65	29.95	27.47	28.27	**30.67**

**Table 3:** PSNR values of our proposed HSR model and other comparison models after image restoration with pixels missing rate 60%.

	Pixels Missing = 60%							
Images	SALSA	BPFA	GSR	JPG-SR	GSRC-NLP	IR-CNN	IDBP	HSR
Bahoon	26.78	25.82	27.74	28.14	28.31	26.53	27.83	**29.37**
Barbara	24.57	28.05	36.42	35.72	36.59	29.67	28.73	**37.00**
Butterfly	26.79	26.06	31.09	31.15	31.46	29.32	30.03	**32.57**
Corn	27.75	27.54	31.39	30.84	31.18	28.89	29.94	**32.34**
Cowboy	26.99	26.36	29.49	29.59	29.99	28.70	28.97	**32.50**
Fence	25.45	27.74	33.23	33.14	33.51	30.46	31.25	**34.32**
Girl	27.02	26.68	29.47	29.87	30.17	28.60	29.32	**31.71**
Leaves	26.29	25.19	33.39	32.82	33.34	29.88	31.74	**35.06**
Lena	31.49	32.38	33.54	35.44	35.95	33.95	33.93	**36.91**
Mickey	27.41	25.75	31.10	31.12	31.29	29.78	31.18	**32.74**
Mural	26.66	25.17	29.98	30.15	30.33	29.16	29.79	**31.30**
Nanna	26.94	26.14	30.13	30.37	30.59	28.95	29.51	**32.05**
Starfish	29.09	28.76	32.89	32.37	32.71	30.41	31.79	**33.68**
Average	27.17	27.05	31.53	31.59	31.96	29.56	30.31	**33.19**

**Table 4:** PSNR values of our proposed HSR model and other comparison models after image restoration with pixels missing rate 50%.

	Pixels Missing = 50%							
Images	SALSA	BPFA	GSR	JPG-SR	GSRC-NLP	IR-CNN	IDBP	HSR
Bahoon	27.98	27.13	29.42	29.61	29.75	27.99	29.23	**32.19**
Barbara	25.66	31.12	39.14	37.79	38.77	31.95	31.57	**39.58**
Butterfly	28.52	28.16	32.78	32.83	33.02	31.08	32.44	**35.28**
Corn	29.39	29.78	33.77	32.94	33.78	31.26	31.61	**35.40**
Cowboy	28.59	28.18	31.69	31.94	31.90	30.79	31.40	**35.65**
Fence	27.25	29.92	35.01	34.62	34.99	32.31	33.24	**36.61**
Girl	28.60	28.46	31.93	31.77	31.95	30.57	31.09	**34.84**
Leaves	28.11	28.13	35.86	35.21	35.79	32.62	34.34	**38.20**
Lena	33.08	34.15	37.64	37.18	37.64	35.71	36.25	**39.54**
Mickey	28.98	27.43	33.86	33.35	33.58	32.24	33.14	**35.87**
Mural	28.20	27.20	31.73	31.72	31.88	30.71	31.35	**33.92**
Nanna	28.53	28.17	32.16	32.21	32.36	31.71	31.35	**35.02**
Starfish	30.90	30.87	34.94	34.31	34.61	32.46	33.81	**36.53**
Average	28.75	29.13	33.50	33.85	34.19	31.65	32.37	**36.04**

**Table 5:** SSIM values of our proposed HSR model and other comparison models after image restoration with pixels missing rate 80%.

	Pixels Missing = 80%							
Images	SALSA	BPFA	GSR	JPG-SR	GSRC-NLP	IR-CNN	IDBP	HSR
Bahoon	0.6040	0.6328	0.6892	0.6629	0.6925	0.6785	0.6606	**0.7154**
Barbara	0.6782	0.6287	**0.9334**	0.8989	0.9242	0.7953	0.7522	0.9305
Butterfly	0.8161	0.6688	0.9223	0.9195	0.9269	0.8880	0.8901	**0.9386**
Corn	0.7103	0.7106	0.8822	0.8574	0.8717	0.8185	0.8430	**0.8843**
Cowboy	0.7965	0.6589	0.8807	0.8668	0.8823	0.8507	0.8407	**0.8989**
Fence	0.6339	0.6236	0.8862	0.8644	0.8817	0.8179	0.8013	**0.8896**
Girl	0.7196	0.6782	**0.9014**	0.8178	0.8381	0.8022	0.7979	0.8581
Leaves	0.7695	0.6576	0.9452	0.9364	0.9412	0.9037	0.9126	**0.9510**
Lena	0.8425	0.8108	0.9249	0.9062	0.9227	0.8831	0.8821	**0.9282**
Mickey	0.8000	0.6512	0.8816	0.8696	**0.9197**	0.8482	0.8422	0.8963
Mural	0.6785	0.6046	0.8158	0.7915	0.8135	0.7766	0.7615	**0.8286**
Nanna	0.7494	0.6721	0.8533	0.8395	0.8552	0.8204	0.8120	**0.8722**
Starfish	0.7594	0.7024	0.8691	0.8516	0.8653	0.8175	0.8286	**0.8787**
Average	0.7352	0.6693	0.8758	0.8525	0.8719	0.8231	0.8173	**0.8823**

**Table 6:** SSIM values of our proposed HSR model and other comparison models after image restoration with pixels missing rate 70%.

	Pixels Missing = 70%							
Images	SALSA	BPFA	GSR	JPG-SR	GSRC-NLP	IR-CNN	IDBP	HSR
Bahoon	0.7024	0.7305	0.7796	0.7597	0.7818	0.7714	0.7456	**0.8012**
Barbara	0.7580	0.8032	**0.9628**	0.9474	0.9579	0.8883	0.8680	0.9606
Butterfly	0.8838	0.8281	0.9506	0.9475	0.9530	0.9326	0.9329	**0.9569**
Corn	0.8624	0.8492	0.9295	0.9142	0.9224	0.8978	0.8969	**0.9298**
Cowboy	0.8742	0.8265	0.9232	0.9127	0.9237	0.9079	0.8952	**0.9341**
Fence	0.7512	0.7726	0.9230	0.9069	0.9203	0.8877	0.8840	**0.9272**
Girl	0.8250	0.8021	0.9014	0.8861	0.8991	0.8792	0.8685	**0.9106**
Leaves	0.8726	0.8209	0.9743	0.9664	0.9697	0.9531	0.9500	**0.9754**
Lena	0.8576	0.9022	0.9507	0.9393	0.9499	0.9277	0.9239	**0.9544**
Mickey	0.8621	0.8097	0.9248	0.9142	0.9240	0.9060	0.9002	**0.9297**
Mural	0.7917	0.7532	0.8743	0.8565	0.8718	0.8551	0.8338	**0.8819**
Nanna	0.8369	0.8030	0.9076	0.8973	0.9075	0.8895	0.8697	**0.9177**
Starfish	0.8675	0.8466	0.9184	0.9032	0.9141	0.8876	0.8839	**0.9227**
Average	0.8266	0.8114	0.9169	0.9040	0.9150	0.8911	0.8810	**0.9232**

**Table 7:** SSIM values of our proposed HSR model and other comparison models after image restoration with pixels missing rate 60%.

	Pixels Missing = 60%							
Images	SALSA	BPFA	GSR	JPG-SR	GSRC-NLP	IR-CNN	IDBP	HSR
Bahoon	0.7756	0.7990	0.8446	0.8264	0.8439	0.8355	0.8140	**0.8709**
Barbara	0.8191	0.8879	**0.9765**	0.9657	0.9744	0.9305	0.9170	0.9757
Butterfly	0.9191	0.8974	0.9666	0.9620	0.9671	0.9535	0.9513	**0.9715**
Corn	0.9022	0.9092	0.9543	0.9442	0.9509	0.9375	0.9290	**0.9599**
Cowboy	0.9064	0.8946	0.9497	0.9413	0.9499	0.9417	0.9273	**0.9613**
Fence	0.8222	0.8523	0.9470	0.9342	0.9445	0.9229	0.9145	**0.9525**
Girl	0.8754	0.8764	0.9359	0.9252	0.9354	0.9227	0.9090	**0.9474**
Leaves	0.9173	0.9064	0.9849	0.9800	0.9833	0.9737	0.9696	**0.9869**
Lena	0.9283	0.9341	0.9668	0.9584	0.9664	0.9499	0.9448	**0.9705**
Mickey	0.8977	0.8717	0.9480	0.9392	0.9472	0.9356	0.9301	**0.9547**
Mural	0.8483	0.8337	0.9086	0.8944	0.9072	0.8972	0.8781	**0.9199**
Nanna	0.8823	0.8707	0.9383	0.9292	0.9382	0.9267	0.9164	**0.9490**
Starfish	0.9036	0.8997	0.9453	0.9336	0.9430	0.9260	0.9209	**0.9514**
Average	0.8767	0.8795	0.9436	0.9334	0.9421	0.9272	0.9171	**0.9517**

**Table 8:** SSIM values of our proposedSR model and other comparison models after image restoration with pixels rate 50%.

	Pixels Missing = 50%							
Images	SALSA	BPFA	GSR	JPG-SR	GSRC-NLP	IR-CNN	IDBP	HSR
Bahoon	0.8357	0.8508	0.8924	0.8781	0.8898	0.8834	0.8640	**0.9287**
Barbara	0.8651	0.9367	0.9850	0.9765	0.9839	0.9562	0.9471	**0.9852**
Butterfly	0.9432	0.9340	0.9759	0.9719	0.9762	0.9671	0.9662	**0.9820**
Corn	0.9310	0.9447	0.9719	0.9640	0.9692	0.9621	0.9514	**0.9783**
Cowboy	0.9344	0.9322	0.9668	0.9599	0.9663	0.9618	0.9518	**0.9770**
Fence	0.8705	0.9048	0.9627	0.9524	0.9605	0.9467	0.9046	**0.9700**
Girl	0.9108	0.9199	0.9581	0.9492	0.9569	0.9497	0.9365	**0.9699**
Leaves	0.9444	0.9534	0.9909	0.9786	0.9901	0.9847	0.9821	**0.9928**
Lena	0.9474	0.9525	0.9779	0.9701	0.9771	0.9649	0.9626	**0.9816**
Mickey	0.9243	0.8932	0.9661	0.9670	0.9645	0.9563	0.9506	**0.9728**
Mural	0.8876	0.8932	0.9345	0.9242	0.9340	0.9275	0.9118	**0.9509**
Nanna	0.9173	0.9202	0.9589	0.9504	0.9577	0.9505	0.9392	**0.9705**
Starfish	0.9335	0.9363	0.9634	0.9541	0.9615	0.9512	0.9458	**0.9714**
Average	0.9112	0.9209	0.9619	0.9536	0.9606	0.9509	0.9395	**0.9716**

**Table 9:** Comparison of running time in seconds of different methods.

Methods	SALSA	BPFA	GSR	JPG-SR	GSRC-NLP	IR-CNN	IDBP	HSR
Time	1.81	1200.23	923.24	499.48	5027.67	9.24	20.33	5000.22

## Data Availability

Datasets used in this research were cited through citing papers corresponding to them. The datasets can be found at the following websites. SALSA: http://sedumi.ie.lehigh.edu. BPFA: http://www.ee.duke.edu/~mz1/Results/BPFAImage/. GSR: https://github.com/jianzhangcs/GSR. JPG-SR: https://drive.google.com/open?id=1KMIERcJtZYKdGt2HvUySFtviAC5RprHu. GSRC-NLP: https://drive.google.com/open?id=1jWtRQ9mUxVzBR0pec. IR-CNN: https://github.com/cszn/IRCNN. IDBP: https://github.com/tomtirer/IDBP. The Kodak photoCD dataset: http://r0k.us/graphics/kodak/.
